# Child-to-Parent Violence and Dating Violence Through the Moral Foundations Theory: Same or Different Moral Roots?

**DOI:** 10.3389/fpsyg.2020.597679

**Published:** 2021-01-08

**Authors:** Maria L. Vecina, Jose C. Chacón, Raúl Piñuela

**Affiliations:** ^1^Department of Social Psychology, Complutense University of Madrid, Madrid, Spain; ^2^Methodology of Behavioral Sciences, Complutense University of Madrid, Madrid, Spain

**Keywords:** moral foundations, child-parent violence, dating violence, juvenile violence, authority (thesaurus)

## Abstract

The objective of this study is to explore and to verify the utility of the five moral foundations (care, fairness, loyalty, authority, and purity) to differentiate between two understudied groups, namely, young offenders who use violence against their parents or dating partners, as well as to predict the extent to which these young people justify violence and perceive themselves as aggressive. Although both types of violence imply, by definition, harming someone (low care) and adopting a position of authority (high authority), we hypothesize a very different role for at least these two moral foundations. Our results support this idea and show a much lower regard for the five moral foundations, including care and authority, in the child-to-parent violence group (CPV; *N* = 65) than in the dating violence group (DV; *N* = 69). Additionally, the authority foundation was able to increase the effectiveness of correctly classifying the participants in one group or the other by 29%. Finally, care and authority, along with fairness, served to predict justification of violence and self-perceived aggressiveness. The moral foundations approach provides preliminary evidence to better understand two specific types of youth violence and extract preventive educational and treatment strategies.

## Introduction

Child-to-parent violence (CPV) and dating violence (DV) are early and apparently modern manifestations of violence and are especially alarming because the violent behavior manifests during the evolutionary development of the individual. There are many theoretical and practical approaches that have attempted to provide explanations for the different manifestations of violence that essentially involves harming someone (Baumeister, 1996; Herrenkohl et al., 2000). One of them has been related to power and authority (Lips, 1991; Rudman and Glick, 2008). Traditionally, most social systems have given power to men over women and parents over children (Wilson and Daly, 1992), and it is precisely in that direction that the majority of violence has been directed throughout history (Pinker, 2011). Only recently, and in the context of the so-called WEIRD societies (Western, educated, industrialized, rich, and democratic; Henrich et al., 2010), has the exercise of authority been called into question, and possibly, as a consequence of this, violence against children and violence against women have been decreasing worldwide (Straus and Gelles, 1986; Pinker, 2011). However, we see in Western countries that violence against women resist to completely be eradicated despite the efforts invested, while violence against parents, which has traditionally been anecdotal, grows every day as a social problem.

Although neither of these two types of youth violence has been studied under the prism of the moral foundations theory (MFT; Haidt, 2007; Graham et al., 2011), it seems quite clear that in both cases the moral foundation of harm is involved, as is the exercise of authority beyond what is considered the norm in our society. In this paper, we explore the moral foundations that are important for two groups of violent youth, CPV and DV, as well as the ability of the moral foundations to differentiate between them and to predict criterion variables used in current psychological treatments (justification of the use of violence and self-perception of aggressiveness). This novel approach may broaden our understanding of this social problem through new variables, unexplored by the current literature on juvenile violence, and thus help us develop prevention and treatment programs based on them.

### Two Types of Youth Violence With the Same or Different Moral Roots?

Child-to-parent violence seems to occur within the family and, contrary to common sense, it is the children, boys, and girls, who exercise violence against the natural authority of those who must guide and educate them (Cottrell and Monk, 2004; Gallagher, 2004; Walsh and Krienert, 2009; Routt and Anderson, 2011; Moulds and Day, 2017; Moulds et al., 2018). Dating violence occurs in the first dating relationships, and violence is exerted by one member in the couple, who asserts an authority over the other, with whom there are no strong commitments, no relationship of coexistence, no children in common, and no binding legal or economic relationships (Shorey et al., 2008; Foshee et al., 2009; Rubio-Garay et al., 2017; Leadbeater et al., 2019).

While the prevalence of child-to-parent violence has increased in the last decade to become a growing social problem (Moulds et al., 2016, 2018; Gallego et al., 2019), intimate partner violence is an old problem, present in different versions in all cultures throughout the history of mankind (Garcia-Moreno et al., 2005; Buss and Duntley, 2011). It seems that the prevalence of this kind of violence among youth and young adults (dating violence) exceeds 20% (Hickman et al., 2004; Niolon et al., 2015; Jennings et al., 2017; Wincentak et al., 2017), and given its severe sequelae for health (Campbell, 2002), the issue of dating violence has moved to the forefront of public health (Vagi et al., 2013).

Recently, the MFT has proposed that perceptions of what is right or wrong may be based on concerns other than care and fairness and has opened the spectrum of morality to other moral foundations, such as ingroup or loyalty, authority, and purity (Haidt, 2007; Haidt and Graham, 2007; Haidt and Joseph, 2008). The first two moral foundations are primarily focused on providing and protecting the rights and freedoms of individuals. These moral concerns are called the “individualizing foundations” and are characterized as follows: (1) care or distaste for the pain of others and (2) fairness or sensitivity to issues related to equality, justice, and rights. The three other moral foundations have a more controversial role and they have been related to idealistic violence and inter-group conflicts (Haidt and Graham, 2007; Graham and Haidt, 2012; Koleva et al., 2012). These are called the binding foundations and they focus on preserving the group as a whole by ties of loyalty, hierarchy, and common beliefs. They are characterized as follows: (3) ingroup, or the tendency to form coalitions and show loyalty; (4) authority, or the propensity to manifest hierarchical social interactions to preserve order within the group; and (5) purity, or the propensity to exhibit emotions of disgust in response to various biological and social contaminants. On top of these five moral foundations, people, groups, and societies create unique moralities by emphasizing different foundations to varying degrees.

It has been proposed that, at least theoretically, two of these moral foundations are breached in any violent act, since a violent act implies harming someone (care) and acting based on hierarchical social structures of dominance and subordination (authority) (Vecina et al., 2015). Empirically, recent studies have connected the five moral foundations with the violent behavior of adult men against their partners, and four different combinations of the moral foundations have been identified among them (Vecina and Chacón, 2019): “sacralizers,” who score highly on the five moral foundations; “all for one,” who score highly on the binding foundations, especially ingroup; “moral outsiders,” with very low scores in every moral foundation; and “purists,” who score highly on care, fairness, and purity. In a similar sample, it was also concluded that not paying enough attention to the care and fairness foundations, while simultaneously holding the authority and ingroup foundations in high regard, can provide a solid basis for upholding sexist attitudes (Vecina and Piñuela, 2017).

Although all the moral foundations have the potential to be relevant in explaining differences between violent young offenders, the care and authority foundations may be key to understand the deep differences between CPV and DV. In this respect, CPV has been defined by two criteria that can be linked directly to the care and authority foundations: (1) causing psychological, physical, or financial harm and (2) engaging in intentional acts to control the parents (Cottrell, 2001). DV has also been equally characterized by the same two elements: (1) the intentional provocation of real harm, whether physical, psychological, or sexual, and (2) the control or dominance of an individual by the partner through threats or coercive tactics (Rubio-Garay et al., 2015). However, and because it is not the same to usurp the legitimate authority of parents, who naturally must have more than their children, as it is to impose an illegitimate authority against a partner, who has equal rights and obligations in our current social system, we argue that this apparent similarity may rely on a different configuration of the moral foundations, where regard for care and authority could be lower in the CPV group than in the DV group. This lower moral profile in the CPV group may be more dangerous because it directly threatens the foundations of the current social order, in which parents rule in order to educate their children in essential restrictions that aim to promote cooperation and prevent harm to others.

A true commitment to the moral foundation of authority involves subjecting one’s authority to limits, that of superiors, in a hierarchy recognized by all parties (Haidt and Joseph, 2004). Such an adaptive strategy for the social order seems to be absent in young people who use violence to impose their will against their parents’ ineffective attempts to impose norms. These young people seem to pursue their individual and personal goals and claim their freedom and personal autonomy, without accepting the very limits that the foundation of authority represents: duty, order, and respect for legitimate authorities as parents, teachers, police, and so on. That fits best with amorality and selfishness or a pre-conventional morality stage (Kohlberg, 1978).

On the contrary, the violence of young men against their young female partners can be understood as an early exercise of men’s authority over women, which makes sense under the traditional systems still in force in many countries and until recently in Western countries as well. These conservative social systems appeal to hierarchies not only where a man prevails over a woman but also where parents prevail over children, leaders over followers, bosses over employees, and so on. Current equality-based normative systems seek to overcome this inequality and qualify as sexist the exercise of authority by men. Despite the efforts being made in Western countries to socialize the new generations in gender equality, a considerable percentage of young people engage in asymmetric relationships in which caring for a partner may coexist with the exercise of controlling authority, leading to paradoxical states of unfairness and harm.

### Objective and Hypotheses

The general objective of this study is to further our knowledge of two specific types of violence through the five moral foundations by answering this research question: Can the five moral foundations be used to differentiate two types of youth violence (DV and CPV), depending on the relevance attributed to them, as well as predict relevant criterion variables for the treatment of young offenders? Specifically, we argue that based on the MFT (Haidt and Joseph, 2004), there is a clear difference between the violence of the CPV participants, who were condemned for harming and not respecting the legitimate authority of their parents, and the violence of the DV participants, who were condemned for trying to impose their authority on someone deemed equal in our society. In the first case, young people dynamite the legitimate social system in which parents educate, or what is the same, limit the autonomy of their children, so as to socialize them in the existing norms. This kind of violence may have more serious consequences and reflect a kind of amorality similar to that found in samples of adult men convicted of intimate partner violence, called “moral outsiders” (Vecina and Chacón, 2019), and in psychopathic profiles (Walsh and Krienert, 2009). In our study, such dangerousness could be reflected in greater justification of violence and even in a greater self-perception as aggressive persons.

In the case of dating violence, it is argued that these young people intend to impose an illegitimate authority that the current social system does not grant them. It is not that they do not care about the care foundation, but rather that they may have to sacrifice it if the authority they believe they have over women is threatened. These exploratory objectives are articulated through the following hypotheses:

H1: There will be significant differences between the CPV group and the DV group at least in the care and authority foundations: those who used violence against their parents will demonstrate a lower regard for the care and authority foundations than those who used violence against their partner. There will be also significant differences between the groups in the criteria variables: the CPV group will perceive themselves as more aggressive and they will justify violence to a greater extent.

H2: At a minimum, the care and authority foundations will serve to correctly classify participants in their respective groups.

H3. At a minimum, the care and authority foundations will be relevant predictors of justification of the use of violence and self-perception of aggressiveness in both groups of young offenders.

## Materials and Methods

### Participants

The participants were 136 young and violent individuals who had been sentenced in court for various violent acts either against their parents (CPV group) or against their young partners (DV group). None of them had been diagnosed with a psychiatric disorder. The study was approved by the university research ethics committee and by the penitentiary institution that had custody of the violent youths who made up the sample. Participation was voluntary and all data were collected under anonymous conditions. The participants, or their parents if they were underage, were asked by the research team to take part in this research project under conditions of anonymity. This is how we guaranteed, first, independence between the psychological treatment and the research and, second, their freedom to decide not to participate in the study. All participants or their parents signed an informed consent. Two cases were removed due to having 50% or more missing data, yielding 134 valid participants.

The CPV group was made up of 65 young offenders in court-mandated treatment and living in two different treatment centers. Their offenses can be considered serious because they met the criteria of having repeatedly used physical violence over time against one of their parents. Thirty-seven were boys (58%) and 28 were girls (42%). The mean age was 16 (*SD* = 1.15), with ages ranging from 14 to 20. Most were Spanish (71%), followed by Latin American (14%), European (7%), and other nationalities (7%). Fifty percent of the participants had finished intermediate-level studies (71%) and 14% only had a basic education. No gender differences involving the five moral foundations and the criterion variables were found in this CPV group. The analyses will thus consider the entire group of boys and girls.

The DV group was made up of 69 young male offenders attending court-mandated psychological treatment in lieu of prison, since they had no prior criminal record. Although there is no consensus on the definition of dating violence, having a common judicial sentence guarantees that the violence exerted by the participants in the sample was similar and considerably serious. The average age of this group was 25 (*SD* = 3.75), with ages ranging from 18 to 29. This age range is appropriate to consider because it includes the periods of middle and late adolescence (Gutgesell and Payne, 2004). Fifty percent of the participants were Spanish, followed by Latin American (41%) and other nationalities (9%). Most of them had finished intermediate-level studies (73%) and 27% only had a basic level of education. None of them was married. Although young women can also perpetrate dating violence, as reflected in the literature (Archer, 2000; Dutton, 2007; Hettrich and O’Leary, 2007), this violence may be anecdotal, since we could find no psychological treatment group for women.

### Instruments

The participants answered the short version of the Moral Foundations Questionnaire (Graham et al., 2011), which measures the degree to which individuals value culturally constructed virtues and concerns, built on each foundation through the Relevance and the Judgments subscales. The Relevance subscale contains two items from each of the five moral foundations (scale from 0 = *not at all relevant* to 5 = e*xtremely relevant*), e.g., “Whether or not someone suffered emotionally” for care and “Whether or not some people were treated differently from others” for fairness. The Judgment subscale also contains two items from each moral foundation (scale from 0 = *strongly disagree* to 5 = *strongly agree*), e.g., “I am proud of my country’s history” for Loyalty, “Respect for authority is something all children need to learn” for authority, and “I would call some acts wrong on the grounds that they are unnatural” for purity. Cronbach’s alpha values were low, but never lower than those reported by Graham et al. (2011). This is acceptable, considering how each scale contains only two items and the authors of the scales relied on heterogeneity to increase confidence that the foundation was maximally represented, instead of resorting to internal consistency via item redundancy.

In order to assess violent attitudes without arousing much social desirability, we asked participants how much they agree with the following sentence: “Sometimes you have to resort to violence if you don’t want others to think you are dumb.” They indicated their answer on a 6-point scale, ranging from 0 (*fully disagree*) to 5 (*fully agree*). This measure may be indicative of the justification to use violence.

In order to evaluate their sense of their own aggressiveness, the participants indicated how accurately the adjective “aggressive” described them on a 5-point Likert scale (0 indicating “never true for me” and 4 indicating “always true for me”). Accounting for the influence of biases on self-perception, we regard their answers as an accurate measure of their self-perceived aggressiveness.

### Data Analysis

Three sets of data analysis have been used to test the hypotheses. First, comparisons of means allow us to determine possible differences in moral foundations between the groups (H1). A logistic regression analysis will then test the ability of the moral foundations to classify participants into one group or another (H2). Finally, a set of linear regression analyses will explore the utility of moral foundations as predictors of two closely related dependent variables: justification of violence and self-perception of aggressiveness. The data were analyzed with R version 3.5.1 (R Core Team, 2018) and the psych (Revelle, 2018) and DescTools (Signorell et al., 2019) packages.

## Results

As hypothesized (H1), there were statistically significant differences between the two groups of violent young participants, CPV and DV, in the care and authority foundations, with the CPV group scoring lower on care and authority than the DV group. These differences were medium and large, respectively, as evidenced by the effect size of more than half a standard deviation (see Table 1). In addition, there were significant differences in the remaining three moral foundations, such that the CPV group also gave less importance to fairness, ingroup, and purity than the DV group.

**TABLE 1:** Descriptors and *t*-tests for variables in the CPV and DV groups.

	**Child–parent violence**			**Dating violence**					
	**Mean**	**SD**	**α**	**Mean**	**SD**	**α**	***T***	***P***	***d***
Care	3.54	0.90	0.649	3.99	0.79	0.566	−3.01	0.003	−0.52
Fairness	3.63	0.94	0.701	3.95	0.69	0.367	−2.19	0.031	−0.38
Ingroup	3.25	0.95	0.400	3.77	0.96	0.565	−3.08	0.003	−0.54
Authority	2.23	1.07	0.542	3.18	1.11	0.639	−5.03	<0.001	−0.87
Purity	2.62	1.05	637	3.18	1.02	0.523	−3.12	0.002	−0.54
JustViol	2.20	1.85		0.68	1.27		26.3 (*)	<0.001 (*)	0.96
Aggressive	2.92	0.98		1.41	1.32		34.6 (*)	<0.001 (*)	1.31

All variables ranged 0–5 except Aggressiveness, ranging 0–4. α, Cronbach’s α; *d* Cohen’s *d* effect size. (*) For Justification of Violence and Aggressiveness, statistics and *p* values come from the Kruskal–Wallis rank sum test, as the *t*-test is not appropriate; see the details the Supplementary Material.

Also as hypothesized, there were even larger differences in the *justification of violence* and *self-perception as an aggressive person* criteria variables. The CPV group scored much higher than the DV group, meaning they justified the use of violence much more and consistently perceived themselves as more aggressive. The shapes of the distributions showed very different patterns (see Supplementary Material). Sixty-nine percent of the DV participants were grouped in the minimum value of the *justification of violence* variable, versus 29% in the CPV group. Similarly, most of the participants in the DV group (55%) exhibited low scores in the *self-perception as an aggressive person* variable (positive asymmetry), compared to 8% of the participants in the CPV group (negative asymmetry).

Regarding hypothesis 2, the results from the logistic regression confirmed that authority, but not care, was a significant predictor of belonging to the groups. Table 2 shows the model, with authority as the sole predictor. This model was chosen after comparing it with two others: the null model, which served as a starting point, and the full model, which includes the five moral foundations (see Supplementary Material). A comparison between the models indicated that the best model was the one that includes only authority as a predictor, as the full model did not provide a significant gain in the reduction of residual deviance. The AIC and BIC indicators show the same preference. For this analysis, 12 participants (9%) were removed due to missing data in the predictors. A reanalysis using multiple imputations yielded virtually identical results (see Supplementary Material for details).

**TABLE 2:** Logistic regression model where authority predicts belonging to the CPV group or the DV group.

**(*n* = 122)**	**β**	**SE**	***z***	***P***
**Predictors**				
Intercept	−2.0702	0.5480	−3.778	<0.001
Authority	0.7991	0.1884	4.241	<0.001

The coefficients of model 1 indicate that when the value for authority is zero, the ratio of cases in the CPV group to DV is exp(β0) = 7.93. That is, for every case of DV, there are 7.83 cases of CPV. The coefficient β1 is negative, which indicates that the probability of being in the CPV group decreases as the value for authority increases. Regarding the precision in the classification, it was observed that it increases from 52.5 to 66.4% when using the authority variable.

Regarding hypothesis 3, two regression analyses for each group of young offenders were performed, one on justification of using violence and another on self-perception of aggressiveness. We followed the same strategy as above, specifying a null model, then a model of interest, and finally a full model that considers all the moral foundations in the equations. Table 3 shows the results of both regressions on authority (see Supplementary Material for details).

**TABLE 3:** Regression of justification of violence and aggressiveness for both the CPV and DV groups.

**Justification of violence**					**Self-perception of aggressiveness**				
**(*n* = 58)**	**β**	**SE**	***T***	***p***	**(*n* = 64)**	**β**	**SE**	***t***	***p***
**CPV**					**CPV**				
Intercept	3.316	0.534	6.21	<0.001	(Intercept)	3.902	0.265	14.708	<0.001
Authority	−0.472	0.218	−2.17	0.034	Authority	−0.424	0.108	−3.943	<0.001
*R*^2^ = 0.078	*R*^2^_*adj*_ = 0.061	*F*(1,56) = 4.72; *p* = 0.034			*R*^2^ = 0.224	*R*^2^_*adj*_ = 0.206	*F*(1,55) = 15.55; *p* ≤ 0.001		
**DV**					**DV**				
(Intercept)	2.914	0.865	3.367	0.001	(Intercept)	1.995	1.181	1.688	0.098
Care	0.575	0.250	2.303	0.025	Care	0.492	0.312	1.574	0.122
Fairness	−1.145	0.277	−4.137	0.000	Fairness	−0.632	0.347	−1.823	0.075
*R*^2^ = 0.223	*R*^2^_*adj*_ = 0.197	*F*(2,61) = 8.75; *p* ≤ 0.001			*R*^2^ = 0.074	*R*^2^_*adj*_ = 0.033	*F*(2,46) = 1.83; *p* = 0.173		

In the CPV group, the authority foundation was the only variable that helped to explain the variance in both justification of violence and self-perception of aggressiveness. The negative weight of the coefficient showed that a low regard for the authority foundation was related to a greater justification of violence and, consistently, to a greater self-perception of aggressiveness. The percentage of variance explained by authority was high for self-perception of aggressiveness and low for justification of violence, 21% and 6%, respectively.

In the DV group, care and fairness were relevant to explaining the justification of violence, but not the self-perception of aggressiveness, such that a high regard for the care foundation and a low regard for the fairness foundation seemed to explain the justification of violence. The percentage of variance explained by care and fairness was high (20%). The absence of significant weights among the five foundations to explain the self-perception of aggressiveness variable allows us to only partially confirm hypothesis 3.

## Discussion

The two types of youth violence analyzed in this paper are relevant social problems, with one growing (violence against parents) and the other persisting (violence against the dating partner). Based on recent research connecting the five moral foundations and intimate partner violence (Vecina and Piñuela, 2017; Vecina and Chacón, 2019), we generally hypothesize that the five moral foundations could also be relevant to portray two types of youth violence, that perpetrated against parents (CPV) and that exercised against the dating partner (DV). These new connections would not only serve to reinforce the applicability of the moral foundations theory to understand controversial attitudes and immoral behaviors but also help to broaden preventive and intervention strategies for these types of violence. This is especially relevant in a context where the effectiveness of interventions points toward short-term effects that decay over time (Gondolf, 2011; Stith et al., 2012; Jennings et al., 2017).

Child-to-parent violence and dating violence can be similarly read through the moral foundations of care and authority because they imply, by definition, harming someone and adopting a position of authority. However, our intuition and common sense say that it is quite different and even more serious to usurp the authority of the parents, who legitimately exercise it in order to educate their children, than to claim an authority over a dating partner that is merely residual in our current social system. In this respect, the young people who harm their parents did not learn from them to respect authority, so they show little regard for this moral foundation to the point that they observe no restrictions, not even to avoid harming their own parents. This idea was translated into a profile with lower scores for the care and authority foundations for the CPV group than for the DV group. It was also hypothesized that at least these two moral foundations would serve to correctly classify a significant percentage of the participants in their respective group and would have the potential to predict external criteria that are relevant for psychological treatments.

Consistently, a central conclusion that can be drawn from the results is that the two groups of violent young people (CPV and DV) seem to differ on all the moral foundations and, especially, on authority and care as hypothesized. The five moral foundations were much less important for those who used violence against their parents than for those who used violence against their partners. Regarding the role of the moral foundations to distinguish between the two groups of young people, it can be concluded that only the authority foundation was a significant predictor, such that as the authority score decreases, the probability of being classified in the CPV group increases. This variable improved the correct classification of the participants by a significant 29%. Finally, and regarding the utility of the care and authority foundation to predict relevant variables inside each group, it can be concluded that in the CPV group, a low regard for the authority foundation was related to both a higher justification of violence and higher self-perception of aggressiveness, while a high regard for care and a low regard for fairness significantly predicted a higher justification of violence in the DV group. The unexpected positive weight of the care foundation is discussed later.

A broader view portrays the group that used violence against their parents as potentially more dangerous than the group that used violence against their partners. They were younger, they show a low regard for all the moral foundations (care, fairness, loyalty, authority, and purity), and they even perceive themselves as much more aggressive and justify the use of violence more. If these violent young people do not care about anything, they have no qualms about pursuing their whims without any limit, no matter how immoral the consequences are. This could be understood as a risk configuration, similar to the profile of “moral outsiders” identified by Vecina and Chacón (2019) and to sociopathic profiles (Heide, 1995; Vaughn and Howard, 2005; Walsh and Krienert, 2009).

The DV participants demonstrated a significantly high regard for all the moral foundations, which makes us think of a risk configuration similar to the moral profile found in adult men convicted of intimate partner violence, called “sacralizers” (Vecina and Chacón, 2019). These young participants seem to care profoundly about both the individualizing foundations that protect individuals and the binding foundations that protect group interests. Conflictive situations can make them prioritize some foundations to the detriment of others within a social system that has remnants of sexism. The positive relationship between the care foundation and the justification for violence, which was not hypothesized but appeared in linear regression, can support this interpretation, as well as the result that none of the moral foundations predicted self-perception of aggressiveness in the DV group. For these young people who used violence against their dating partners could coexist in the same cocktail and without apparent contradiction something like that: “I care about my dating partner and I can impose the authority I think I have even if that is unfair and harms her.”

All these exploratory results reflect a promising field of new research with larger samples and experimental approaches that would allow connecting the well-established moral foundations theory and different risk profiles for various types of violence and extracting lessons for education and socialization.

### Study Limitations and Applications

The exploratory nature of this research and its cross-sectional approach do not allow us to draw definitive conclusions. Thus, no causal inferences can be made from this study. However, it is the first research that explores the five moral foundations in such sensitive samples of violent young people with condemnatory judicial sentences for different violent crimes. Nevertheless, and because this study constitutes a first attempt to explore CPV and DV in the MFT domain, a descriptive and comparative approach could be considered cautious. The results do support theoretical ideas and empirical data using ecological samples and may be considered sufficiently consistent to provide a solid basis for new studies. They point to some peculiar moral roots for different violent behaviors and how the authority foundation seems to differentiate them.

A more specific limitation refers to the wide range of ages of the two groups. This was unavoidable since the groups were under mandated psychological treatment in different centers and for different crimes, consistent with their age: residential for the CPV group of minors and non-residential for the DV group older than 18. It could be also said that the age, much lower in the group of young people who harm their parents, could explain all the differences found in this research, and this may indeed be so. However, age alone is not a variable that helps treat problems, while different configurations of the moral foundations may help to design more effective approaches to prevention and intervention. In this respect, our results have the potential to advance our understanding of the multiple causes of violence in truly problematic samples.

Thinking of young offenders who used violence against either their partners or their parents as having peculiar configurations of the five moral foundations may allow psychologists to incorporate new strategies that focus, for example, on increasing or decreasing the importance of certain moral foundations. Although it seems quite clear that all of them harm someone, some may be doing it because they have little respect for the restrictions of the moral foundation of authority and others because they have too much. In the first case, it could be useful to reinforce the authority of parents and educational figures in our current social system. Their greater experience and development makes them the wisest option to address the uncertain future of new and still maturing generations. In the second case, there is an indisputable need to continue promoting equality and fair treatment between women and men, since current achievements in gender equality have not arisen spontaneously, but through multiple and wide-ranging efforts. However, it could be useful to add new elements to the current campaigns to reduce sexist attitudes, such as the clarification of values related to care, fairness, and authority, and to set priorities in case of conflict in favor of the first two.

## Data Availability Statement

The datasets presented in this study can be found in online repositories. The names of the repository/repositories and accession number(s) can be found in the article/Supplementary Material.

## Ethics Statement

The studies involving human participants were reviewed and approved by the Comisión de Calidad de la Facultad de Psicología de la UCM. Written informed consent to participate in this study was provided by the participants’ legal guardian/next of kin.

## Author Contributions

All authors listed have made a substantial, direct and intellectual contribution to the work, and approved it for publication.

## Conflict of Interest

The authors declare that the research was conducted in the absence of any commercial or financial relationships that could be construed as a potential conflict of interest.
