# Supplementary material for: Child-to-Parent Violence and Dating Violence Through the Moral Foundations Theory: Same or Different Moral Roots?
Source: Front Psychol. 2021 Jan 8;11:597679. doi: 10.3389/fpsyg.2020.597679 (PMC7874107; doi:10.3389/fpsyg.2020.597679)
Supplement: Supplementary file 1 [file Table_1.docx]

**Supplementary Material**

We include here some additional information about variables properties, treatment of missing data and complementary analyses.

**Missing data**

Initial data were composed by 136 cases, but two were deleted due the number of missing data (four and six missings), so the analyses were carried out on 134 cases (106 cases have no missing data, 27 cases have 1 missing data, and one case has 2 missing data). Table 1 shows the number of missing data per variable for these 134 participants.

Table 1. Missing data for the eigth variables used in this study

|  | n | TipViol | Care | Fairn | Ingr | Auth | Purit | JustVi | Aggre | Tot |
| --- | --- | --- | --- | --- | --- | --- | --- | --- | --- | --- |
| CPV | 65 | 0 | 2 | 1 | 1 | 1 | 2 | 0 | 1 | 8 |
| DV | 69 | 0 | 0 | 1 | 3 | 0 | 2 | 0 | 15 | 21 |
| n miss | 134 | 0 | 2 | 2 | 4 | 1 | 4 | 0 | 16 | 29 |
| % miss |  | 0 | 1.5 | 1.5 | 3 | 0.7 | 3 | 0 | 11.9 |  |

*Note.* CPV, Children-Parent Violence group; DV, Dating violence group.

MFQ dimensions has less than 5% of missing data, and imputation is not necessary (Graham, 2009; Schafer, 1999) when analyzing individual variables (i.e., in descriptives and *t* tests for Moral Foundations). But in regression analyses, cases with any missing data are deleted and the number of valid cases can be reduced up substantibly. So, imputations procedures are recommended in these cases.

We used the R package “mice” (van Buuren & Groothuis-Oudshoorn, 2011), based on multivariate imputation by chained equations (MICE; Azur, Stuart, Frangakis & Leaf, 2011; Raghunathan et al., 2001; Van Buuren, 2007). Package mice assumes missing at random (MAR), and a careful review of the questionnaires with missing values (especially for Aggressivity) did not show any pattern or circumstance that could be related to the missing data. Using mice, values were imputed through chained equations followed by predictive mean matching (PMM), as it is the combination which yielded the best results in previous simulation studies (Marshall, Altman, & Holder, 2010; Marshall, Altman, Royston, & Holder, 2010). Imputed sets were analyzed separately and results were pooled using the Rubin’s (1987) rules, including some diagnostic statistics (as the fraction of missing information, *fmi*). The recommended number of imputed sets may vary from five to 10 in early studies to, more recently, 40 sets for increasing power (see, for instance, Graham, Olchowski, & Gilreath, [2007](https://www.ncbi.nlm.nih.gov/pmc/articles/PMC3074241/#mpr329-bib-0009)). Given the small size of our data and the low proportion of missing data, we decided to generate 20 sets.

After analyzing imputed data, numeric results differ just slightly, and all the conclusions remain identical. The variable Self-perceived Aggressiveness was omitted from imputation, as recommended for dependent variables in regression (von Hippel, 2007). So results shown in the main text are relative to the original data.

**Moral Foundations: Exploratory analysis and group comparisons**

Apart from the centrality and dispersions measures in the main text, below are the statistics and *p*-values for normality test (Shapiro-Wilks), skewness and kurtosis, and the *p*-value for Levene test for homogeneity of variance. All calculations were made using the R software, version 3.5.1 (R Core Team, 2018).

Table 2. Shapiro-Wilks test, skewness, kurtosis and homogeneity test of variance for the Five Moral Foundations in groups CPV and DV

|  | CPV | | | |  | DV | | | |  | Levene |
| --- | --- | --- | --- | --- | --- | --- | --- | --- | --- | --- | --- |
|  | S-W | *p* | Skew | Kurt |  | S-W | *p* | Skew | Kurt |  | *p* |
| Care | 0.94 | .003 | -1.00 | 2.14 |  | 0.93 | .001 | -0.70 | -0.06 |  | .514 |
| Fairness | 0.94 | .005 | -0.79 | 0.47 |  | 0.95 | .011 | -0.30 | -0.57 |  | .148 |
| Ingroup | 0.96 | .023 | -0.41 | -0.48 |  | 0.93 | .001 | -0.77 | 0.06 |  | .547 |
| Authority | 0.98 | .225 | 0.04 | -0.79 |  | 0.97 | .141 | -0.36 | -0.13 |  | .977 |
| Purity | 0.89 | <.001 | -0.99 | 0.25 |  | 0.96 | .017 | -0.67 | 0.66 |  | .835 |

*Note.* CPV, Children-Parent Violence group; DV, Dating violence group; S-W, Shapiro-Wilks.

The graphics below show the distribution for the Moral Foundations divided by groups, and were obtained from the R package DescTools (Signorell et mult. al., 2019). It can be seen Authority is the moral foundation showing greater differences.

Figure 1. Density and box plots of Moral foundations by Type of violence

| 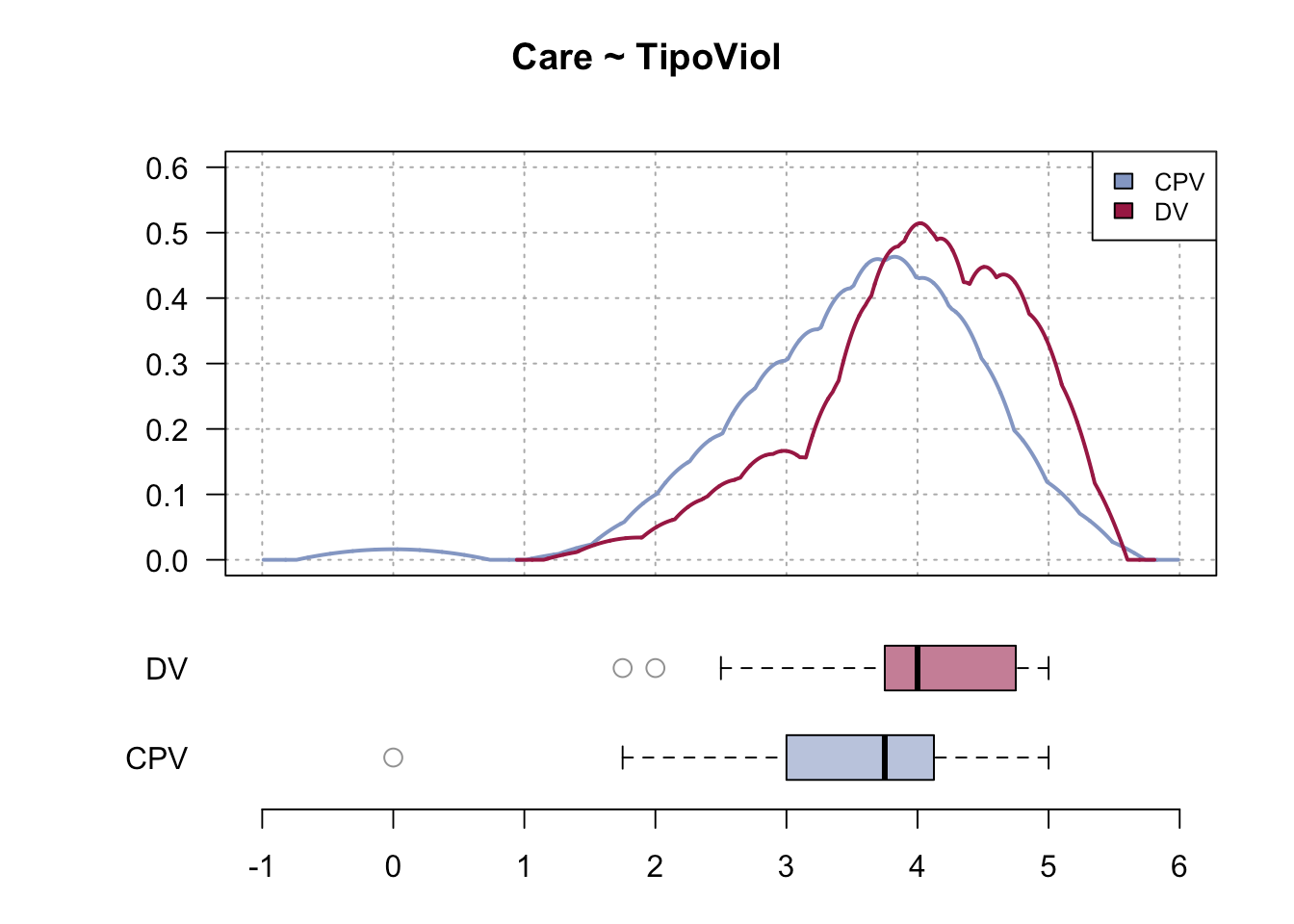 | 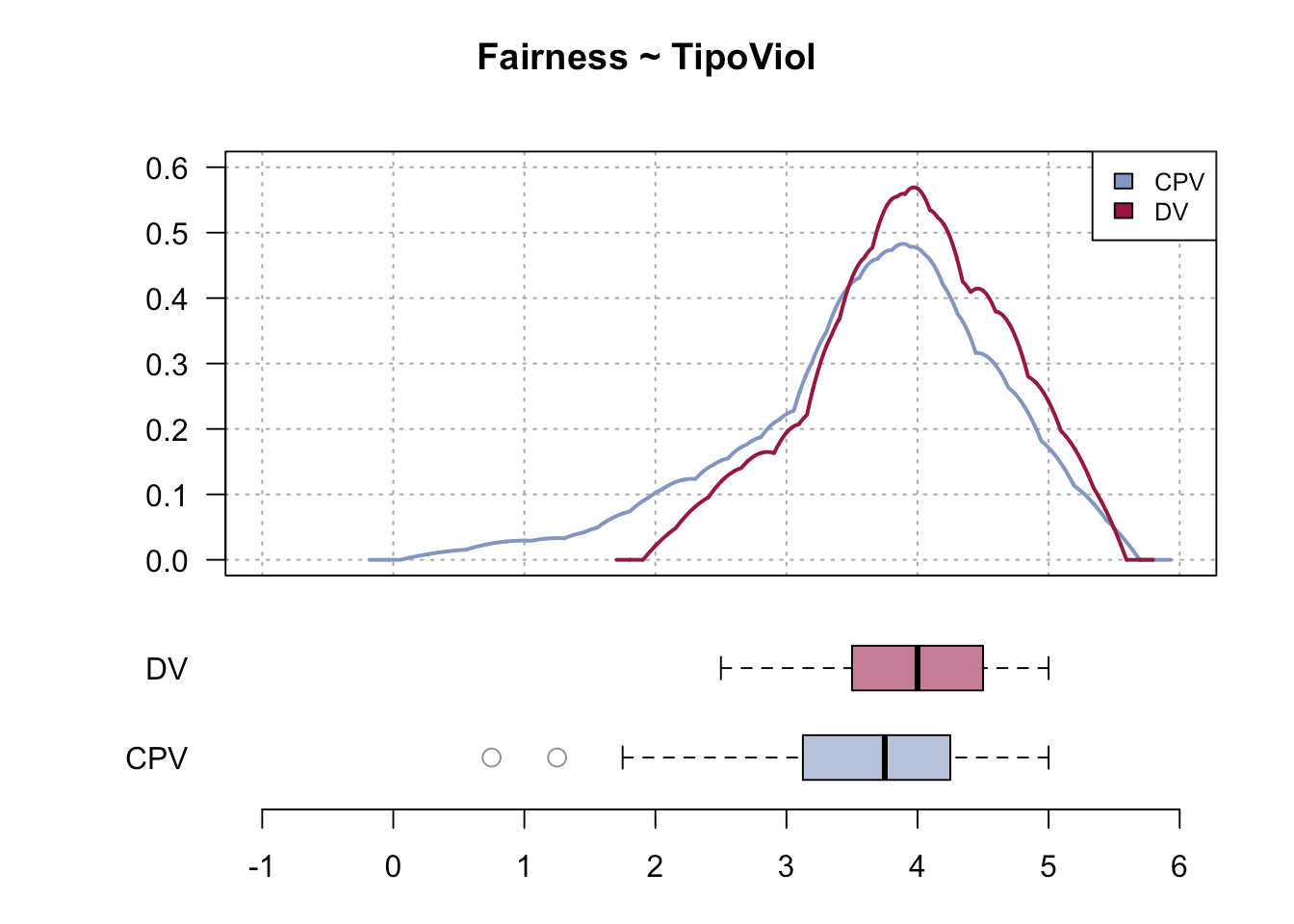 |
| --- | --- |
| 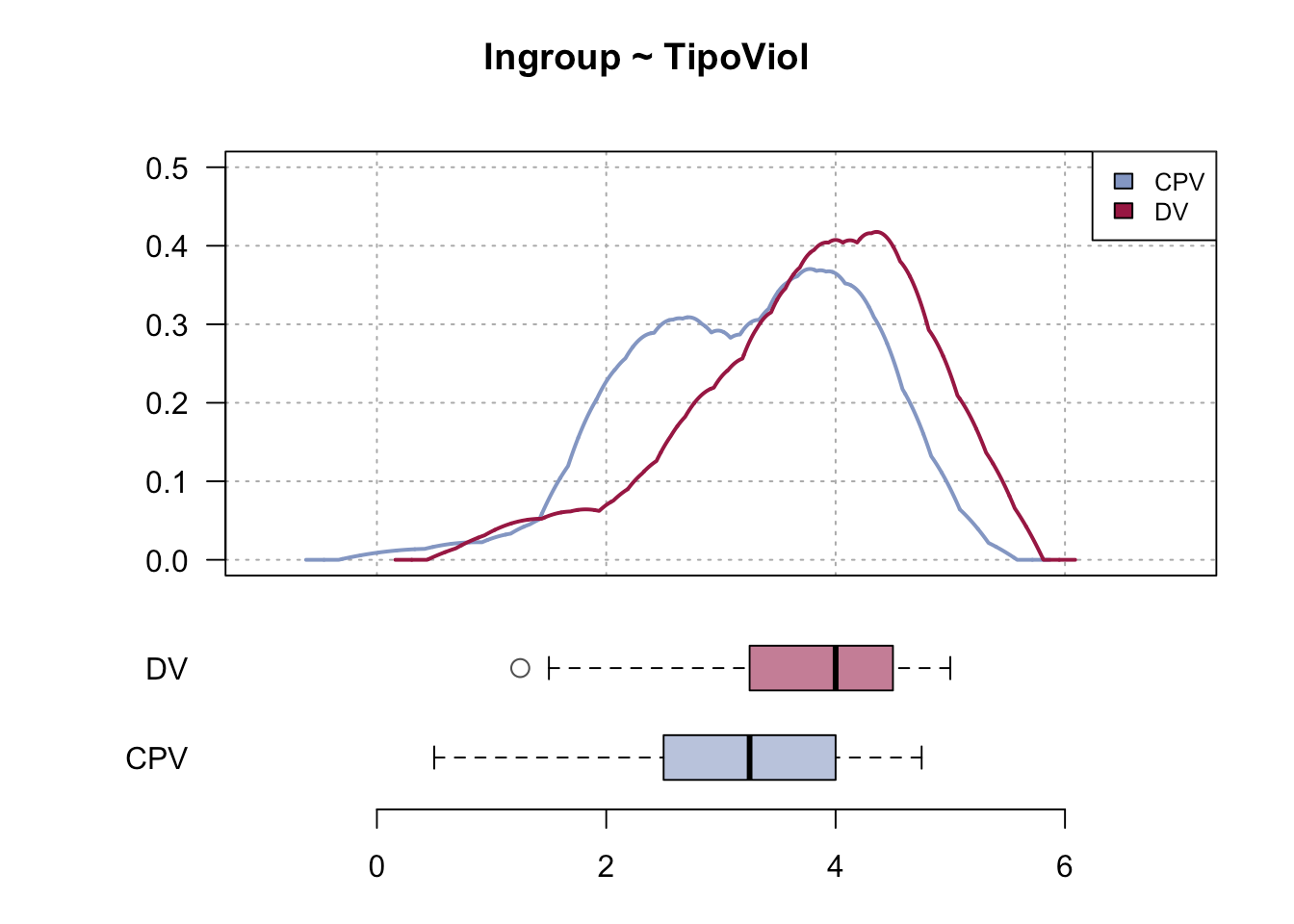 | 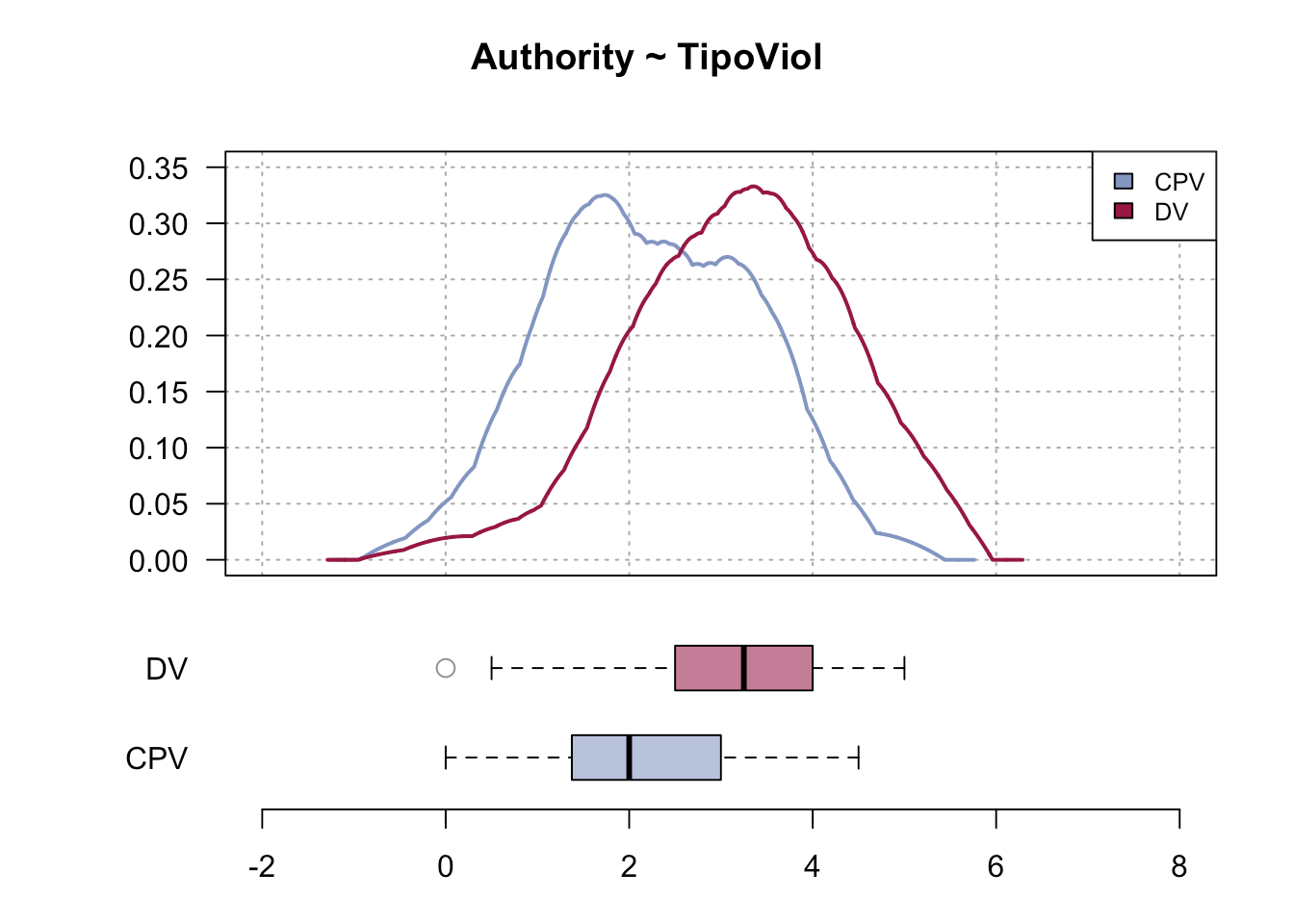 |
| 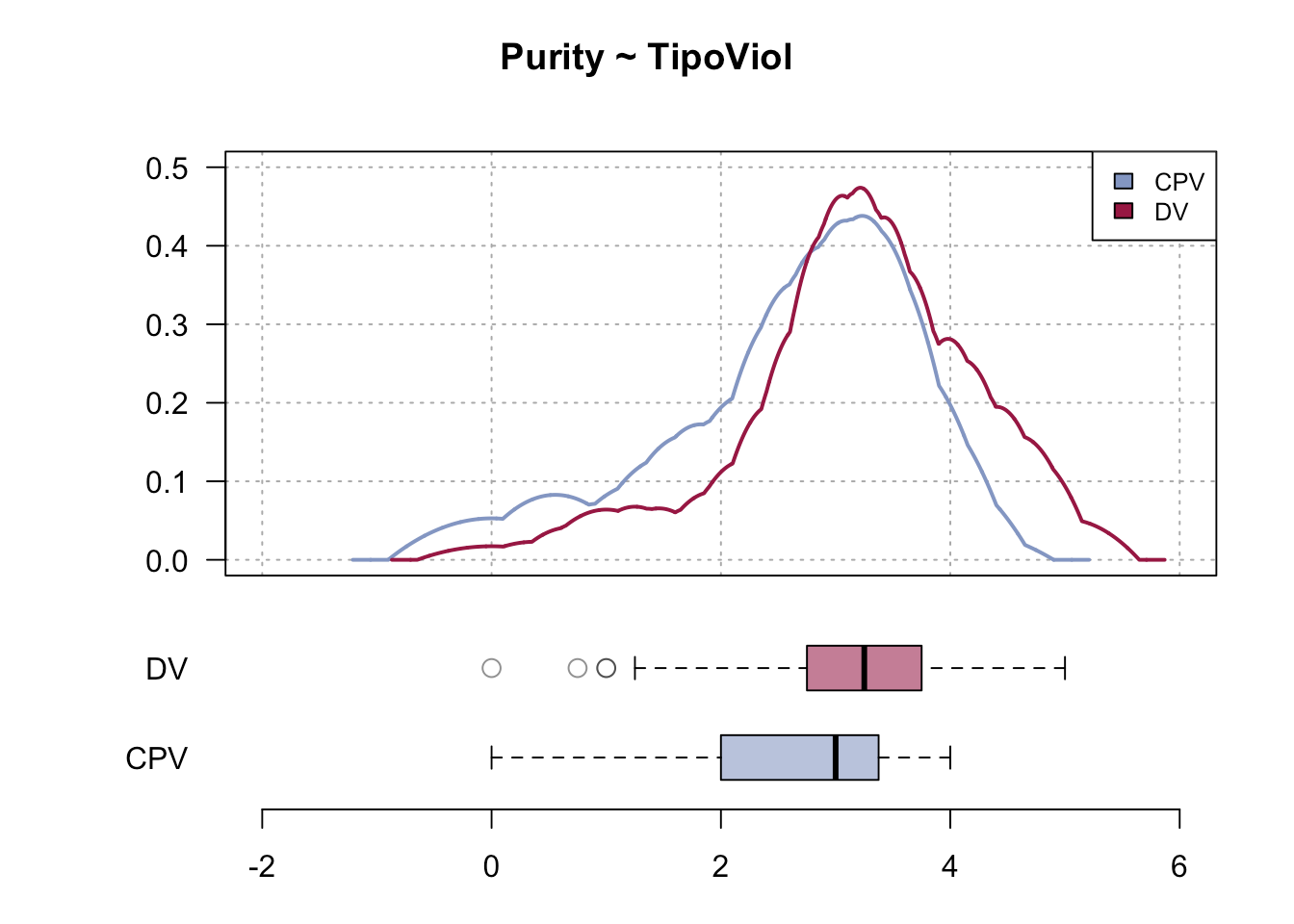 |  |

Of the five Moral Foundations, only Authority fully meets the assumptions for a *t*-test. However, in all other cases no significant differences are found in their between-group variances (all p >= .15 in Levene’s tests) and asymmetries, if any, have the same direction. So, t-tests are appropriate for all Moral Foundations, as showed in Table 1 in main text. Additional Kruskal-Wallis rank sum tests yielded the same results.

**Justification of violence and Aggressiveness: Exploratory analysis and group comparisons**

Regarding the variables Justification of violence and Aggressiveness, below are the analyses of normality, shape and between-groups homogeneity of variances, followed by the variables’ graphical representation.

Table 3. Shapiro-Wilks test, skewness, kurtosis and homogeneity test of variance for variables Justification of Violence and Aggressiveness in groups CPV and DV

|  | CPV | | | |  | DV | | | |  | Levene |
| --- | --- | --- | --- | --- | --- | --- | --- | --- | --- | --- | --- |
|  | S-W | *p* | Skew | Kurt |  | S-W | *p* | Skew | Kurt |  | *p* |
| JustViol | 0.87 | <.001 | 0.14 | -1.46 |  | 0.61 | <.001 | 1.89 | 2.70 |  | <.001 |
| Aggress | 0.84 | <.001 | -0.94 | 0.73 |  | 0.86 | <.001 | 0.58 | -0.78 |  | .007 |

*Note.* CPV, Children-Parent Violence group; DV, Dating violence group; S-W, Shapiro-Wilks.

Figure 1. Density and box plots for Justification of Violence and Self-perceived Aggressiveness by Type of Violence

| 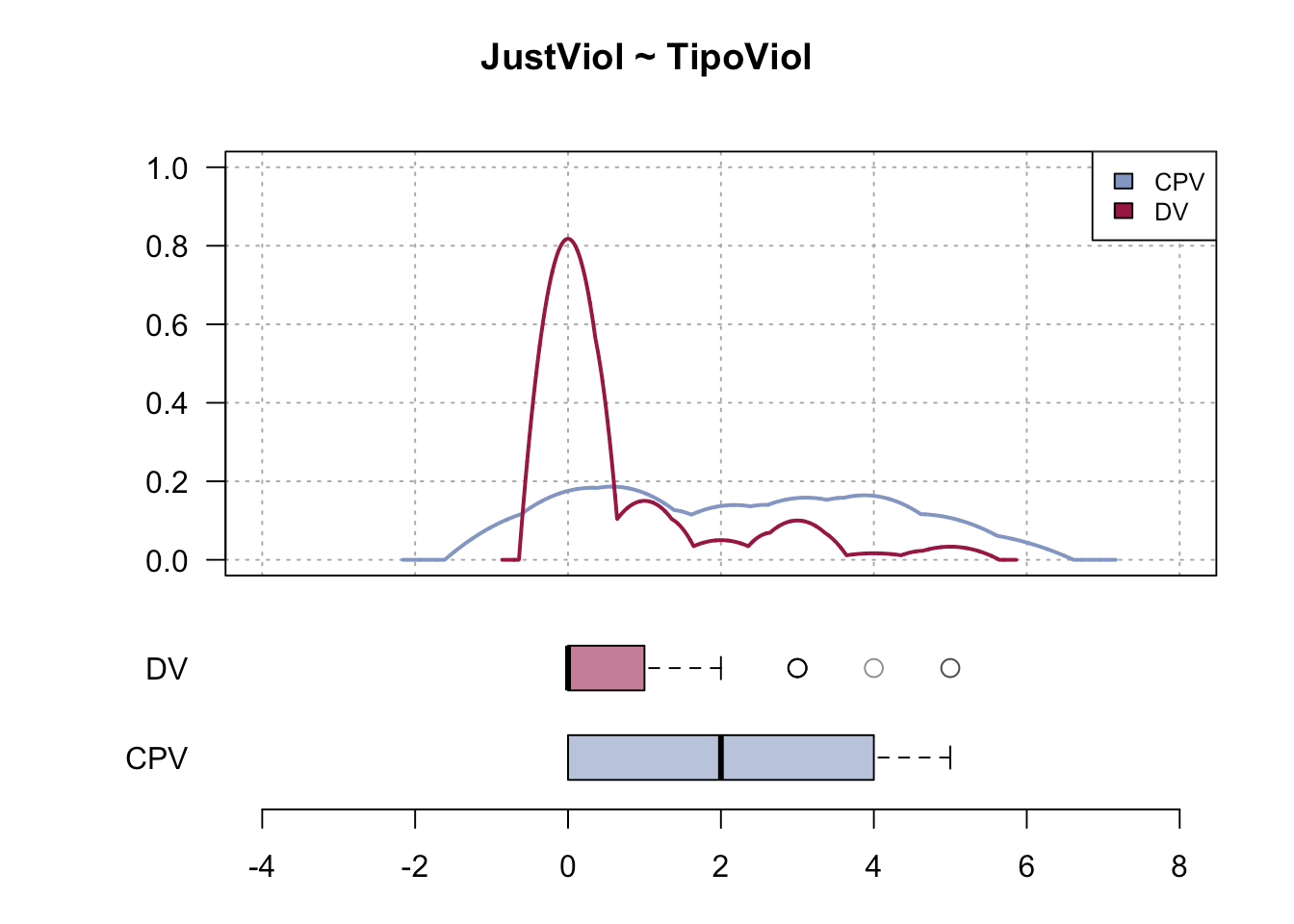 | 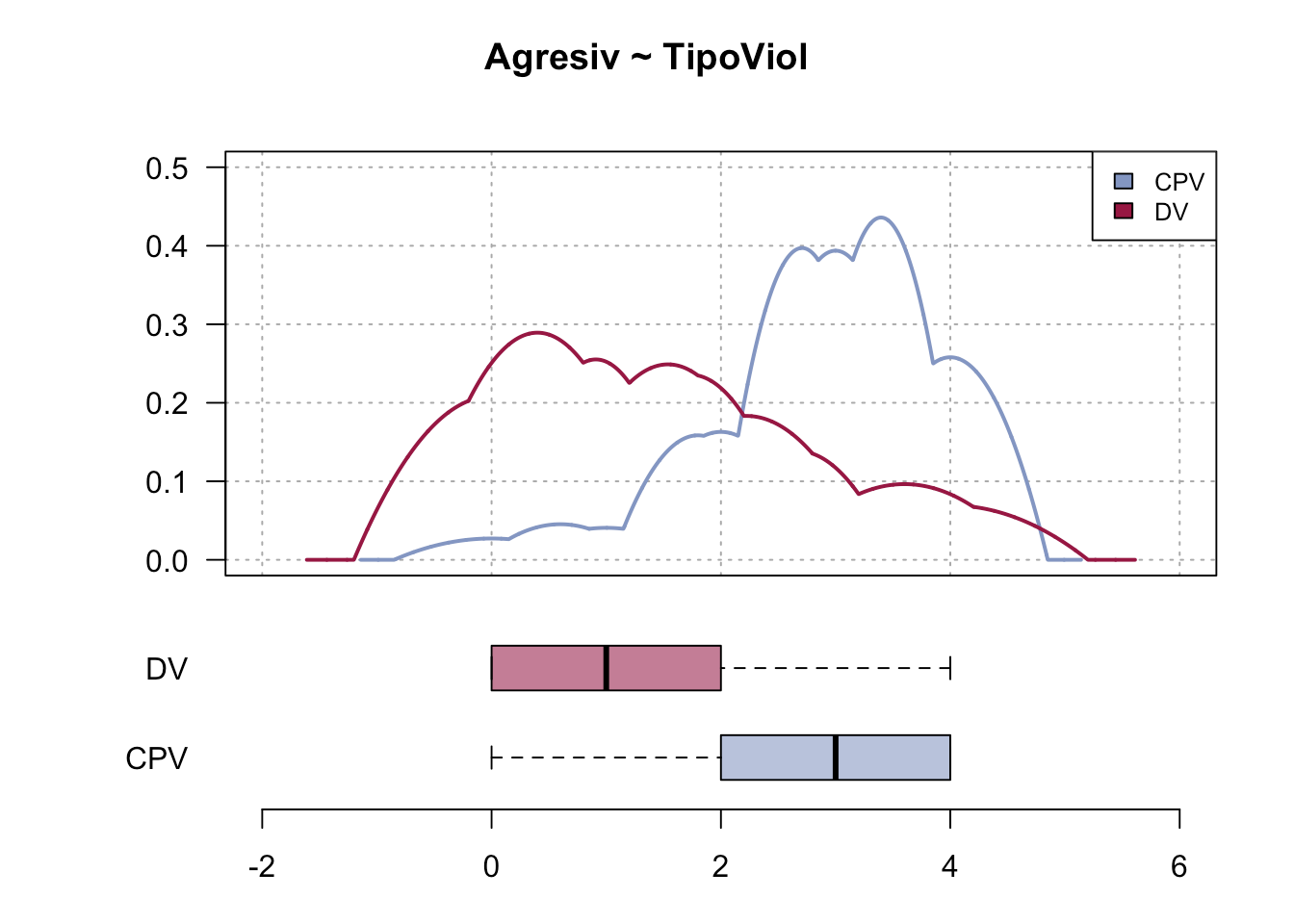 |
| --- | --- |

For these variables, both the statistics and the graphical representations show conditions that recommend using a test that does not depend on these assumptions, such as the Kruskal-Wallis rank sum test, whose results are shown in Table 1 of the main text.

**Logistic regression**

Logistic regression finds the best model to predict a dichotomous variable from a set of predictors. In our case, the question is whether any of the dimensions of the moral foundations is a good predictor of the type of violence perpetrated by the participants and, more particularly, (hypothesis 2) whether the Authority is the only (or best) predictor of the type of violence.

Previously, linearity between predictors and the log-odds of the dependent variable were assessed graphically, showing an adequate linear adjustment. No outliers or influential obserations were found, and multicollinearity can be rejected, as observed in the correlation matrix in Table 4. However, observe that multicollinearity is not appliable in the selected model (Model 1) as it has just one predictor.

Table 4. Correlation matrix for the Moral Foundations predictors used in Logistic Regression Models

|  | Care | Fairness | Ingoup | Authority | Purity |
| --- | --- | --- | --- | --- | --- |
| Care | 1.000 | .644 | .412 | .420 | .375 |
| Fairness | .644 | 1.000 | .291 | .322 | .328 |
| Ingroup | .412 | .291 | 1.000 | .572 | .547 |
| Authority | .420 | .322 | .572 | 1.000 | .595 |
| Purity | .375 | .328 | .547 | .595 | 1.000 |

*Note.* All *p*s were less or equal to .001.

We carry out a study considering three models: the null model, which will serve as a starting point and reference; a model with Authority as the only predictor, and the full model, which includes the five Moral Foundations. In the null model, without predictors, the category estimated for all cases is that with greater frequency.

Table 5. Logistic Regression Models for Type of Violence on the Moral Foundations

|  | Original data (n = 122) | | | |
| --- | --- | --- | --- | --- |
| **NULL model** | β | SE | z | *p* |
| Intercept | -0.0984 | 0.1813 | -0.543 | .587 |
| **Model 1** | β | SE | z | *p* |
| Intercept | 2.0702 | 0.5480 | 3.778 | <.001 |
| Authority | -0.7991 | 0.1884 | -4.241 | <.001 |
| **Full Model** | β | SE | z | *p* |
| Intercept | 3.0547 | 1.2169 | 2.510 | .012 |
| Care | -0.3480 | 0.3248 | -1.072 | .284 |
| Fairness | 0.0897 | 0.3174 | 0.283 | .777 |
| Ingroup | 0.0616 | 0.2583 | 0.239 | .812 |
| Authority | -0.6717 | 0.2325 | -2.889 | .004 |
| Purity | -0.1987 | 0.2502 | -0.794 | .427 |

The results show Authority as the only significant predictor. Table 6 shows the comparisons among the three models, including AIC and BIC indicators. Nagelkerke *pseudo* R^2^ were obtained with R package "rcompanion" (Mangiafico, 2020).

Table 6. Logistic Regression Models for Type of Violence on the Moral Foundations

|  | Residual df | Residual deviance | df | Deviance | *p* | AIC | BIC | *pseudo* R^2^  Nagelkerke | Global | CPV | DV |
| --- | --- | --- | --- | --- | --- | --- | --- | --- | --- | --- | --- |
|  |  |  |  |  |  |  |  |  | Accur. | Accur. | Accur. |
| Original data (n = 122) | | | | | | | | | | | |
| Null Model | 121 | 168.83 |  |  |  | 172.8 | 178.4 |  | 52.46% | 0% | 100.0% |
| Model 1 | 120 | 146.29 | 1 | 22.54 | <.001 | 152.3 | 160.7 | 0.2251 | 66.39% | 62.07% | 70.31% |
| Full Model | 116 | 144.24 | 4 | 2.05 | .726 | 158.2 | 177.9 | 0.2436 | 68.03% | 63.79% | 71.88% |

*Note*. *p* values correspond to tests for a significant increment of explained deviance respect to the previous model.

The three last columns in Table 6 include the global accuracy and that for DV and CPV groups as percentage of correct classifications, which are equivalent to sensitivity and specificity (or viceversa; but not applicable here as there is no reference class). It should be noted that under the null model, the prediction consists only of assigning to all cases the category with the highest proportion of observations, in this case, DV.

**Linear regression**

Hypothesis 3 proposed Authority as the main Moral Foundation to predict Justification of the use of violence and Self-perception of aggressiveness in both groups of violent young people, although other predictors were considered posible, too. As before, we started adjusting the null model, followed by the full model and finally a more well-adjusted model.

Assumptions for the final models were tested, including linearity, homogeneity, normality of residuals and the existence of outliers. All models showed normal residuals and no influential values. Linearity and homogeneity of residuals were found to be met for all models except for the Justification of Violence on Care and Fairness, where diagnostic graphs detected a greater dispersion for high values.

The following tables show the results of the regression for the variables Justification of Violence and Aggressiveness on Moral Foundations.

Table 7. Regression of Justification of Violence and Aggressiveness on Authority for group CPV

| Justification of Violence | | | | | | |  | Self Perception of Aggressiveness | | | | | |
| --- | --- | --- | --- | --- | --- | --- | --- | --- | --- | --- | --- | --- | --- |
| (n = 58) | | β | SE | | *t* | *p* |  | (n = 64) | β | SE | | *t* | *p* |
| **Null model** | |  |  | |  |  |  | **Null model** |  |  | |  |  |
| Intercept | | 2.276 | 0.245 | | 9.31 | <.001 |  | Intercept | 2.965 | 0.132 | | 22.399 | <.001 |
| **Model 1** | |  |  | |  |  |  | **Model 1** |  |  | |  |  |
| Intercept | | 3.316 | 0.534 | | 6.21 | <.001 |  | (Intercept) | 3.902 | 0.265 | | 14.708 | <.001 |
| Authority | | -0.472 | 0.218 | | -2.17 | .034 |  | Authority | -0.424 | 0.108 | | -3.943 | <.001 |
| *R*^2^ = .078 | *R*^2^_adj_ = .061 | | | F(1, 56) = 4.72; *p* = .034 | | |  | *R*^2^ = .224 | *R*^2^_adj_ = .206 | | F(1, 55) = 15.55; *p* = <.001 | | |
| **Full model** | |  |  | |  |  |  | **Full model** |  |  | |  |  |
| Intercept | | 1.750 | 1.214 | | 1.441 | .156 |  | (Intercept) | 3.775 | 0.599 | | 6.305 | <.001 |
| Care | | -0.094 | 0.339 | | -0.278 | .728 |  | Care | -0.126 | 0.167 | | -0.753 | .455 |
| Fairness | | 0.325 | 0.315 | | 1.033 | .306 |  | Fairness | 0.113 | 0.156 | | 0.724 | .473 |
| Ingroup | | 0.376 | 0.325 | | 1.158 | .252 |  | Ingroup | 0.193 | 0.160 | | 1.204 | .234 |
| Authority | | -0.632 | 0.282 | | -2.245 | .029 |  | Authority | -0.364 | 0.140 | | -2.599 | .012 |
| Purity | | -0.063 | 0.318 | | -0.199 | .843 |  | Purity | -0.237 | 0.159 | | -1.489 | .143 |
| *R*^2^ = .125 | *R*^2^_adj_ = .041 | | | F(1, 52) = 1.483; *p* = .211 | | |  | *R*^2^ = .264 | *R*^2^_adj_ = .192 | | F(5, 51) = 3.66; *p* = .007 | | |

*Note*. We used original data as there is only one and two missing data respectively.

Table 8. Comparison of Linear Regression Models in group CPV

| Justification of Violence | | | | | | |  | Self Perception of Aggressiveness | | | | | | |
| --- | --- | --- | --- | --- | --- | --- | --- | --- | --- | --- | --- | --- | --- | --- |
| (n = 122) | Res df | Res SS | df | SS | *F* | *p* |  | (n = 122) | Res df | Res SS | df | SS | *F* | *p* |
| Null Model | 57 | 197.59 |  |  |  |  |  | Null Model | 56 | 55.93 |  |  |  |  |
| Model 1 | 56 | 182.24 | 1 | 15.34 | 4.61 | .036 |  | Model 1 | 55 | 43.60 | 1 | 12.32 | 15.28 | <.001 |
| Full Model | 52 | 172.93 | 4 | 9.32 | 0.70 | .595 |  | Full Model | 51 | 41.14 | 4 | 2.45 | 0.76 | .556 |

Table 9. Regression of Justification of Violence and Aggressiveness on Care and Fairness for group DV

| Justification of Violence | | | | | | |  | Self Perception of Aggressiveness | | | | | |
| --- | --- | --- | --- | --- | --- | --- | --- | --- | --- | --- | --- | --- | --- |
| (n = 64) | | β | SE | | *t* | *p* |  | (n = 64) | β | SE | | *t* | *p* |
| **Null model** | |  |  | |  |  |  | **Null model** |  |  | |  |  |
| Intercept | | 0.703 | 0.162 | | 4.35 | <.001 |  | Intercept | 1.49 | 0.191 | | 7.78 | <.001 |
| **Model 1** | |  |  | |  |  |  | **Model 1** |  |  | |  |  |
| (Intercept) | | 2.914 | 0.865 | | 3.367 | .001 |  | (Intercept) | 1.995 | 1.181 | | 1.688 | .098 |
| Care | | 0.575 | 0.250 | | 2.303 | .025 |  | Care | 0.492 | 0.312 | | 1.574 | .122 |
| Fairness | | -1.145 | 0.277 | | -4.137 | <.001 |  | Fairness | -0.632 | 0.347 | | -1.823 | .075 |
| *R*^2^ = .223 | *R*^2^_adj_ = .197 | | | F(2, 61) = 8.75; *p* = <.001 | | |  | *R*^2^ = .074 | *R*^2^_adj_ = .033 | | F(2, 46) = 1.83; *p* = .173 | | |
| **Full model** | |  |  | |  |  |  | **Full model** |  |  | |  |  |
| Intercept | | 2.931 | 0.898 | | 3.264 | .002 |  | Intercept) | 2.223 | 1.225 | | 1.815 | .076 |
| Care | | 0.578 | 0.272 | | 2.128 | .038 |  | Care | 0.596 | 0.333 | | 1.786 | .081 |
| Fairness | | -1.202 | 0.298 | | -4.033 | <.001 |  | Fairness | -0.559 | 0.387 | | -1.447 | .155 |
| Ingroup | | 0.010 | 0.196 | | 0.053 | .958 |  | Ingroup | -0.216 | 0.246 | | -0.877 | .385 |
| Authority | | -0.132 | 0.173 | | -0.761 | .450 |  | Authority | -0.053 | 0.216 | | -0.247 | .806 |
| Purity | | 0.180 | 0.186 | | 0.969 | .337 |  | Purity | 0.014 | 0.231 | | 0.061 | .952 |
| *R*^2^ = .239 | *R*^2^_adj_ = .173 | | | F(5, 58) = 3.642; *p* = .006 | | |  | *R*^2^ = .099 | *R*^2^_adj_ ~ 0 | | F(5, 43) = 0.94; *p* = .461 | | |

Table 10. Comparison of Linear Regression Models in group DV

| Justification of Violence | | | | | | |  | Self Perception of Aggressiveness | | | | | | |
| --- | --- | --- | --- | --- | --- | --- | --- | --- | --- | --- | --- | --- | --- | --- |
| (n = 122) | Res df | Res SS | df | SS | *F* | *p* |  | (n = 122) | Res df | Res SS | df | SS | *F* | *p* |
| Null Model | 63 | 105.359 |  |  |  |  |  | Null Model | 48 | 86.245 |  |  |  |  |
| Model 1 | 61 | 81.872 | 2 | 23.487 | 8.494 | .001 |  | Model 1 | 46 | 79.905 | 2 | 6.340 | 1.754 | .185 |
| Full Model | 58 | 80.186 | 3 | 1.686 | 0.407 | .749 |  | Full Model | 43 | 77.697 | 3 | 2.208 | 0.407 | .749 |

**Referencias**

Azur, M. J., Stuart, E. A., Frangakis, C., & Leaf, P. J. (2011). Multiple imputation by chained equations: what is it and how does it work?. *International Journal of Methods in Psychiatric Research*, *20*(1), 40–49. https://doi.org/10.1002/mpr.329

Falk, C. & Savalei, V. (2011). The Relationship Between Unstandardized and Standardized Alpha, True Reliability, and the Underlying Measurement Model. *Journal of Personality Assessment, 93,* 445-53. DOI: 10.1080/00223891.2011.594129.

Graham J. W. (2009) Missing data analysis: making it work in the real world. *Annual Review of Psychology, 60*, 549–576, DOI: 10.1146/annurev.psych.58.110405.085530

Graham J. W., Olchowski A. E., Gilreath T. D. (2007) How many imputations are really needed? Some practical clarifications of multiple imputation theory. *Prevention Science, 8*, 206–213, DOI: 10.1007/s11121-007-0070-9

Heymans, M. (2020). *psfmi: Prediction Model Selection and Performance Evaluation in Multiple Imputed Datasets*. R package version 0.2.0. https://CRAN.R-project.org/package=psfmi

Lorincz-Comi, N. (2020). *glmice: A model diagnostic and fit statistic calculator for logit models performed on data sets imputed using 'mice'*. R package version 0.1.0. https://github.com/noahlorinczcomi/glmice.git

Mangiafico, S. (2020). *rcompanion: Functions to Support Extension Education Program Evaluation.* R package version 2.3.25. https://CRAN.R-project.org/package=rcompanion

Marshall, A., Altman, D. G., & Holder, R. L. (2010). Comparison of imputation methods for handling missing covariate data when fitting a Cox proportional hazards model: a resampling study. *BMC Medical Research Methodology*, *10*(1), 112.

Marshall, A., Altman, D. G., Royston, P., & Holder, R. L. (2010). Comparison of techniques for handling missing covariate data within prognostic modelling studies: a simulation study. *BMC Medical Research Methodology*, *10*(1), 7.

Meng, X. L., & Rubin, D. B. (1992). Performing likelihood ratio tests with multiply-imputed data sets. *Biometrika*, *79*(1), 103-111. <https://doi.org/10.1093/biomet/79.1.103>

R Core Team (2018). *R: A language and environment for statistical computing*. R Foundation for Statistical Computing, Vienna, Austria. https://www.R-project.org/.

Raghunathan T. W., Lepkowksi J. M., Van Hoewyk J., Solenbeger P. (2001) A multivariate technique for multiply imputing missing values using a sequence of regression models. *Survey Methodology, 27*, 85–95.

Revelle, W. (2018) *psych: Procedures for Personality and Psychological Research*, Northwestern University, Evanston, Illinois, USA, https://CRAN.R-project.org/package=psych Version = 1.8.12.

Schafer J. L. (1999) Multiple imputation: a primer. *Statistical Methods in Medical Research, 8*, 3–15, DOI: 10.1177/096228029900800102

Signorell, A. *et mult. al.* (2019). *DescTools: Tools for descriptive statistics*. R package version 0.99.30. https://cran.r-project.org/package=DescTools

Van Buuren S. (2007) Multiple imputation of discrete and continuous data by fully conditional specification. *Statistical Methods in Medical Research, 16*, 219–242, DOI: 10.1177/0962280206074463

van Buuren, S., & Groothuis-Oudshoorn K. (2011). mice: Multivariate Imputation by Chained Equations in R. *Journal of Statistical Software, 45*(3), 1-67. URL <https://www.jstatsoft.org/v45/i03/>.

Von Hippel, P. T. (2007). Regression with missing Ys: An improvedstrategy for analyzing multiply imputed data. *Sociological Methodology, 37(1)*, 83–117. DOI: [https://doi.org/10.1111/j.1467-9531.2007.00180.x](https://doi.org/10.1111%2Fj.1467-9531.2007.00180.x)
